# New mutant alleles for Spargel/dPGC-1 highlights the function of Spargel RRM domain in oogenesis and expands the role of Spargel in embryogenesis and intracellular transport

**DOI:** 10.1093/g3journal/jkad142

**Published:** 2023-06-27

**Authors:** Swagota D Roy, Sabarish Nagarajan, Md Shah Jalal, Md Abul Basar, Atanu Duttaroy

**Affiliations:** Biology Department, Howard University, 415 College St. NW, Washington D.C., USA 20059; Biology Department, Howard University, 415 College St. NW, Washington D.C., USA 20059; Biology Department, Howard University, 415 College St. NW, Washington D.C., USA 20059; Biology Department, Howard University, 415 College St. NW, Washington D.C., USA 20059; Biology Department, Howard University, 415 College St. NW, Washington D.C., USA 20059

**Keywords:** *Drosophila*, oogenesis, Spargel, Notch, PGC-1

## Abstract

Energy metabolism in vertebrates is controlled by three members of the PGC-1 (PPAR γ− coactivator 1) family, transcriptional coactivators that shape responses to physiological stimuli by interacting with the nuclear receptors and other transcription factors. Multiple evidence now supports that Spargel protein found in insects and ascidians is the ancestral form of vertebrate PGC-1's. Here, we undertook functional analysis of *srl* gene in *Drosophila*, asking about the requirement of Spargel per se during embryogenesis and its RNA binding domains. CRISPR- engineered *srl* gene deletion turned out to be an amorphic allele that is late embryonic/early larval lethal and Spargel protein missing its RNA binding domain (Srl*^ΔRRM^*) negatively affects female fertility. Overexpression of wild-type Spargel in transgenic flies expedited the growth of egg chambers. On the other hand, oogenesis is blocked in a dominant-negative fashion in the presence of excess Spargel lacking its RRM domains. Finally, we observed aggregation of Notch proteins in egg chambers of *srl* mutant flies, suggesting that Spargel is involved in intracellular transport of Notch proteins. Taken together, we claim that these new mutant alleles of *spargel* are emerging powerful tools for revealing new biological functions for Spargel, an essential transcription coactivator in both *Drosophila* and mammals.

## Introduction

Transcription coactivators lack a DNA binding domain, yet by forming complexes with transcription factors, they can alter the local chromatin structure, facilitate assembly of transcriptional complexes, or bind to the transcription apparatus at promoter sites to regulate transcription activity. Thus, transcription coactivators influence a multitude of cellular, developmental, and physiological processes. In mammals, the Peroxisome Proliferator γ− coactivator (PGC-1) plays critical roles in metabolically active tissues. Many nuclear receptors like PPAR, RXR, ERR, NRF2, NRF1 as well as several transcription factors are activated by PGC-1 to influence essential cellular processes such as mitochondrial biogenesis (Wu *et al*. 1999), oxidative metabolism (St-Pierre *et al*. 2003), adaptive thermogenesis (Puigserver *et al*. 1998), antioxidant defense (St-Pierre *et al*. 2006) and heart development (Lai *et al*. 2008).

In mammals, the PGC-1 gene family consist of PGC-1α, PGC-1β and PGC-1 related coactivator (PRC) (Andersson and Scarpulla 2001; Kressler *et al*. 2002; Lin *et al*. 2002; Lin *et al*. 2003). All three share significant homology in functional domains such as the N-terminal activation domain, which includes a LXXLL signature motif involved in nuclear receptor docking and interaction with other transcriptional coactivators. PGC-1 C-terminal motifs include the RRM (RNA Recognition Motif) and the RS (Serine/Arginine) domain, which is involved in mRNA processing and splicing (Villena 2015). Within the family, PRC is functionally distinct with its limited homology to α and β and highly expressed in proliferating cells. Although PGC-1 family members are expressed in metabolically active tissues such as muscle, brain, heart, and brown fat, both ubiquitous and tissue-specific single knockouts of PGC-1α and PGC-1β exhibit relatively mild phenotypes (Leone *et al*. 2005; Uldry *et al*. 2006; Zechner *et al*. 2010). Even in double knockout mice (PGC-1α and PGC-1β) neither mitochondrial mass (Rowe *et al*. 2013) or muscle fiber composition (Zechner *et al*. 2010) is impacted. In contrast, mice lacking PRC are early lethal (He *et al*. 2012). Thus, the moderate phenotypes of PGC-1α, β and their double mutant are likely explained due to gene redundancy.

In *Drosophila*, Spargel (*srl*) is the only PGC-1 family member, thus offering an excellent paradigm for circumventing the complications of gene redundancy. In fact, Spargel is present in many other invertebrates including mosquito and sea-squirt species (LeMoine *et al*. 2010). Therefore, studying Spargel has potential to gain insight into the ancestral function of the PGC-1 gene family and particularly why it remains conserved in mammals (LeMoine *et al*. 2010). In *Drosophila*, Spargel function is known to be associated with oxidative metabolism given that mRNA expression of mitochondrial OXPHOS genes is reduced in *srl* mutants and mitochondrial O_2_ consumption boosted in flies with Spargel gain-of-function (Tiefenböck *et al*. 2010). Overexpression of Spargel cause increased mitochondrial DNA contents, with elevated activity of citrate synthetase, heat shock protein 60 (Hsp60) (Rera *et al*. 2011) and increased mitochondrial O_2_ consumption (Tiefenböck *et al*. 2010). Through genetic epistasis analysis, Spargel was found to act downstream of TOR, S6K, Tsc, and FOXO in the insulin-Tor signaling pathway (Mukherjee and Duttaroy 2013).

In earlier work, we established that Spargel is prevalently expressed in the ovaries of adult female *Drosophila* where it turned out to be functionally essential for female fertility and ovarian growth (Mukherjee *et al*. 2014; Basar *et al.* 2019. However, to further elucidate the biological role of Spargel we need more mutant alleles of the gene in addition to the existing *spargel* hypomorphic allele (*srl*^*1*^) and few *srl RNAi* mutants. Thus, in this report, we describe a *srl* loss of function mutant, and a mutant *srl* lacking the RRM domain (*spargel*^*ΔRRM*^). A set of transgenic flies are made to overexpress wild type Spargel, SpargelΔRRM and SpargelΔRRM + ΔRS proteins in ovaries. A broad functional relationship between PRC and Spargel has been established from their mutant phenotypes. Finally, intracellular transport of Notch protein is negatively affected in *srl* mutants since coagulates of Notch appears in the cytoplasm of *srl* mutants. Taken together, these new mutant alleles of *spargel* are powerful tools for further dissection of the biological functions of Spargel, an essential transcriptional coactivator in both *Drosophila* and mammals.

## Methods

### Drosophila strains

We obtained the following *Drosophila melanogaster* stocks from Bloomington *Drosophila* Stock Center (BDSC):

1. *w^1118^*, (*w[1118]*); (*w*[*]; *P{w[ + mC] = matalpha4-GAL-VP16}V37*, (P{w[ + mC] = otu-GAL4::VP16.R}1, w[*]; (*P{w[ + mC] = GAL4-nos.NGT}40; P{w[ + mC] = GAL4::VP16-nos.UTR}CG6325[MVD1]*, a.k.a MTD-GAL4 driver).
2. *P{ry[ + t7.2] = hsFLP}1, y[1] w[1118]*; *Dr*[Mio]/*TM3*, *ry*[*] *Sb*[1].
3. w[*]; P{w[ + mC] = matalpha4-GAL-VP16}V37.(a.k.a. MAT-Gal4 driver)
4. *w[1118]; P{w[ + mC] = UAS-GFP.nls}8*
5. P{ry[ + t7.2] = neoFRT}40A, y[1] w[*]
6. *Tor[DeltaP] P{ry[ + t7.2] = neoFRT}40A/CyO*
7. *w[1118]; MKRS, P{ry[ + t7.2] = hsFLP}86 E/TM6B, Tb[1]*

Following *spargel* mutant stocks are generated in Duttaroy lab:

Endogenous *srl* mutants:

1. *w[1118]; srl*^*del*^*(LoxP STOP.RFP LoxP)/*[*TM3, Sb. Ser] = UASp-EGFP)*
2. *w[1118]; srl*^*ΔRRM*^*/TM6, Tb*

Transgenic *srl* mutants:

3. *w[1118]; P{w[ + mC] = UASp-EGFP.srl*^+^*}/CyO)*,
4. *w[1118]; P{w[ + mC] = UASp-EGFP. srl*^*ΔRRM + RS*^*}/CyO)*;
5. *w[1118]; P{w[ + mC] = UASp-EGFP*-*srl*^*ΔRRM*^}/CyO;
6. *w[1118]; P{w[ + mC] = UASp-EGFP. srl*^*ΔRS*^*}/CyO*),

New FRT Line in 81F6:

7. w[1118]; *FRT 81F6 srl {LoxP STOP.RFP LoxP}/TM6. Tb*
8. *w[1118]; FRT 81F6 {FRT}/TM6.Tb*

New FRT stocks for somatic recombination:

9. *P{ry[ + t7.2] = hsFLP}1, y[1] w[1118]*; *FRT 81F6 srl {LoxP STOP.RFP LoxP}/TM3, ry*, Sb
10. w[*]; P{w[ + mC] = matalpha4-GAL-VP16}V37/CyO; *FRT 81F6 {FRT} P{w[ + mC] = UAS- GFP.nls}8/TM6.Tb*
11. w*; P{w[ + mC] = Act5C-GAL4}25FO1/CyO; FRT81F6, UAS-GFP, w+/TM6, Tb.

### Yeast feeding regimen, ovary preparation, and fertility assay

We used the standard yeast/cornmeal agar media to nurture all experimental and control flies at 23°C. For ovary preparation and subsequent fertility assay, two days old female flies are maintained on regular food supplemented with yeast paste for 3 more days to allow for rapid ovarian growth [Drummond-Barbosa and Spradling (2001)].

Ovaries were dissected in Grace's media (Life technologies 11605-094), and fix with freshly prepared 4% formaldehyde, 0.3% Triton X-100 in 1 × PBS at room temperature for 20 minutes on a rotator, then washed 3 × with 1XPBST (1XPBS + 0.3% Triton × 100) for 15 minutes and finally mounted in VECTASHIELD carrying DAPI (Vector Labs).

For egg collection purposes, egg laying cages were set up each with 5 females and 5 males on grape juice agar plates carrying yeast paste. Eggs were counted every 24 hours during the egg laying window. The averages number of eggs per female per day were determined and plotted. Significance was calculated based on an unpaired t-test.

### Generation of *srl* mutants

**Fig. 1.**
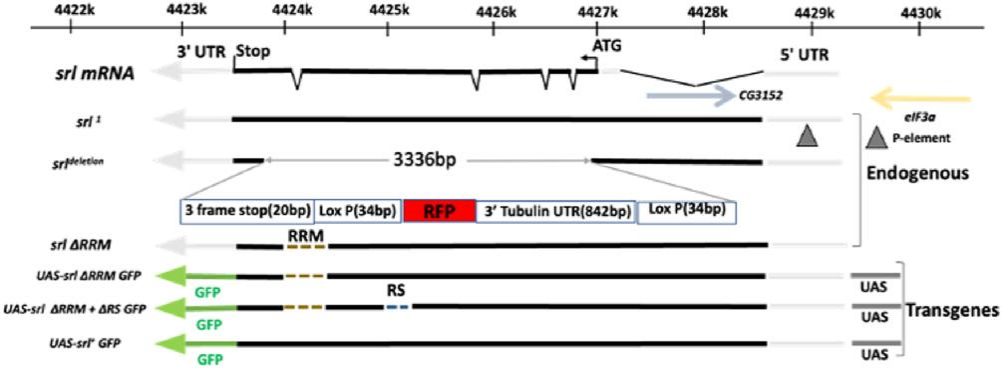
Schematics of various mutants in *spargel* gene. Mutants can be categorized under two classes: endogenous *srl* mutants consists of *srl*^*1*^, *srl*^*del*^, and *srl*^*ΔRRM*^. *srl*^*1*^ allele is described by Tifenbock *et al.* (2010) as a P-element insertion in the *srl* 5′-UTR. In *srl*^*del*^ an exogenous DNA sequence consists of an RFP tag flanked with two loxP sites replace a large part of the *srl* coding region. Second category of mutants carry transgenes capable of expressing domain specific deletions of *spargel* in wild type background when combine with GAL4 driver. All transgenic constructs carry a GFP tag at the C-terminal end which helps in tracing the fusion protein.

Genomic deletions:

1. *Spargel*^*del*^: We generated a 3302-bp deletion within the *srl* locus by targeting two CRISPR target sites (PAM) flanking exon 2 and exon 5. The chosen PAM sites (CAACTG ACAGATACACTGAG[CGG] and TCCAGCGAGATGAACCTAC[CGG]) are complementary to exon 2 and exon 5 of *srl*, respectively. The gRNA1 and gRNA2 were cloned separately into vector 1. A 3XP3- RFP cassette in donor vector provided repair templates for homology- directed repair of the Cas9-induced cleavage sites. The presence of an RFP site flanked by two loxP sites facilitates future excision by Cre recombinase as well as screening of *srl*^*del/+*^ heterozygotes in the F1 generation (Fig. 1. We confirmed the targeted deletion of *srl* exon 2- 5 region by PCR and DNA sequencing. *srl*^*del*^ turned out to be a legitimate loss of function mutation for *srl* and therefore termed as *srl*^*null*^ which is balanced over *TM3, sb, ser-GFP*.
2. Δ*spargel*^ΔRRM^: The RNA recognition motif (322 bp) of endogenous *spargel* (RIVYVG RIEQETTKEILRRKFLPYGSIKQITIHYKENGMKYGFVTYERAQDAFTAIDTSHR DSQISMYDISFGGRRAFCRSSYA) is deleted through CRISPR/Cas9 mediated genome editing. Chosen CRISPR Target Sites [PAM] are:

ATTCGCCGCTCTT CAACGGC[CGG]; and the downstream gRNA 2 CRISPR Target Sites [PAM]: CGTTCATCTT ACGCTGACTT[GGG] was designed and cloned into vector 1. Next steps of amplification, cloning and injection were done as described for *srl*^*null*^. Finally, the ΔRRM deletion was confirmed by PCR and the *srl*^*ΔRRM*^ line was balanced over *TM6, Tb*.

*Transgenic lines:*

1. *UAS-srl*^*+*^*-GFP:* Plasmid carrying *spargel*^*+*^ was tagged with GFP using the Gateway cloning system, injected into the *w1118* background, and expressed under the control of a UAS-sequence.
2. *UAS-srl*^ΔRRM:^ A transgenic line was made to overexpress Spargel protein lacking the RRM and RS domain under control of a UAS-sequence.

*Introducing a new FRT-insertion at FRT81F6* :

The canonical FRT82B6 insertion is located immediately downstream to the *srl* locus. Such closeness hinders mitotic recombination and subsequently clone formation. To alleviate this issue, we used CRISPR to introduce a novel FRT insertion upstream to the *srl* gene at 81F6. The chosen gRNA sequence CGGAATTGCAGAAAACGAGC[TGG] is in the *CG12581* at 81F6 where an FRT site is newly introduced into the long intron of *CG12581* in *w^1118^.* Incidentally, *CG12581* doesn’t express in ovaries so the FRT insertion won’t interfere with somatic recombination during any stage of oogenesis. Complete genotype of the new FRT insertion stock in 3R is *w[1118]; FRT 81F6 {FRT}/TM6.Tb and* w[1118]; *FRT 81F6 srl {LoxP STOP.RFP LoxP}/TM6. Tb*.

### Clonal analysis

To generate *srl*^*null/null*^ egg chambers F1 females (3–5 days old) of the genotype *P{ry[ + t7.2] = hsFLP}1 y[1] w[1118]; P{w[ + mC] = matalpha4-GAL-VP16}V37; FRT 81F6, P{w[ + mC] = UAS-GFP.nls}8/FRT 81F6 srl {LoxP STOP.RFP LoxP}* were generated. Adult females were heat-shocked 3 × per day 30 mins each at 37°C with 2 to 3 hours interval between heat shocking. This regimen was followed for 3 consecutive days. Ovaries were dissected on the 6th day and fixed for immunostaining as described previously (Basar *et al.* 2019). Spargel^−/−^ negative follicle cell clones were generated in F1 females of the genotype ry, hsflp, y, w*/w*; P{w[ + mC] = Act5C-GAL4}25FO1FRT81F6/+; FRT81F6, UASGFP, w^+^/FRT81F6, srl^*null*^. Females were recognized and heat shocked. Dominant negative clones of *Tor* were generated in F1 females of the genotype *w[1118]; P{ry[ + t7.2] = neoFRT}40A. MKRS, P{ry[ + t7.2] = hsFLP}86 E/Tor[DeltaP] P{ry[ + t7.2] = neoFRT}40A.* TorΔP homozygous clones were marked by the absence of GFP.

### Immunostaining, imaging, and antibodies

8–10 pairs of ovaries were dissected in Grace's media (Life technologies 11605-094). All fixation, washings, incubation in primary and secondary antibodies were done following Basar *et al.* (2019). Mouse mAbs used in this study include anti-Spargel (7A10, Duttaroy lab), anti-Delta extracellular domain (DSHB C594.9B), anti-Notch extracellular domain (DSHB C458.2H), anti-Notch intracellular domain (DSHB C17.9C6). We also used anti-rabbit and anti-mouse IgG (TFS Invitrogen R6393) and rabbit anti-GFP (TorreyPines Biolabs). As secondary antibodies, we used Alexa 488-conjugated goat anti-mouse and Alexa 488-conjugated goat anti-rabbit (Cell signaling 4412S).

**Quantification and Statistical analysis:** All measurements were done using NIS Elements Ar imaging software from at least three independent experiments. Data were subjected to parametric student's t-test. Student's t-test was used to assess statistical significance between two groups of data. Statistically significant differences are as follows: ^∗^*P* < 0.05 and ^∗∗∗^*P* < 0.001. For ovarian growth count 45 individual ovaries were used and graphed with Prism 9 and Microsoft excel.

### Microscopy

We visualized and imaged mounted ovaries using a Nikon Ti-E-PFS inverted microscope equipped with a Yokogawa CSU-X1 spinning disk confocal unit. The whole ovary images were taken with Zeiss Axioskop 2 plus microscope which is equipped with PhotoFluor LM-75.

## Results

### A *bona fide spargel* null mutant is late embryonic/early larval lethal

**Fig. 2.**
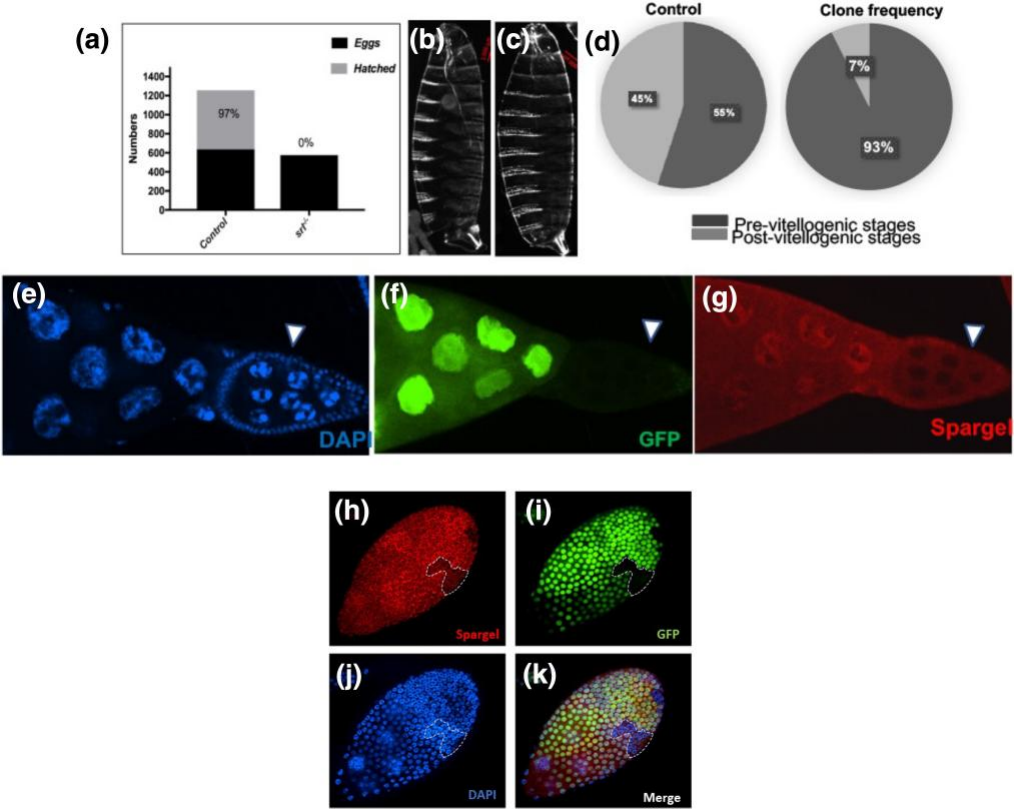
Proof of *srl*^*del*^ as an amorphic mutant. a) Spargel deletion homozygote eggs never hatch with pharate larvae seen inside the egg case. b, c) Normal denticle belts are formed in control and *srl*^*del*^ homozygous embryos. e–f) Clones of homozygous *srl*^*del*^ egg chambers (GFP negative) are also negative for Spargel expression in nurse cells. d) 93% *srl*^*del*^ clones appeared in previtellogenic stage whereas in control it is 55% suggesting the requirements of Spargel in postvitellogenic development. h–k) *srl*^*del*^ cell clones in follicle cells also appeared negative for Spargel expression.

We generated a 3302 base pair deletion of the region between exon 2 to exon 5 within the endogenous *spargel* gene (*srl*^*del*^) (see methods) (Fig. 1). *srl*^*del/del*^ larvae fail to hatch (Fig. 2a) and eventually die as “pharate larvae” (i.e. enclosed inside of the cuticle) with fully formed denticle belts and mouth parts (Fig. 2b and c). Because we observed viable and throbbing larvae inside egg cases until 30 hours after egg laying (see Supplementary Video 1), we concluded that *srl*^*del/del*^ homozygotes are late embryonic/early larval lethal (Fig. 2).

Next, we generated *srl*^*del/del*^ cell clones in nurse cells (Fig. 2e–g) and in follicle cells (Fig. 2h–k), since Spargel is expressed both cell types (Basar *et al.* 2019). Given the proximity of the *srl* locus to the *FRT82B* insertion, the canonical insertion for making somatic clones in 3R, we were unable to use *FRT82B*. So, with CRISPR/Cas9 system (see method for details) we generated a new FRT line in *FRT81F6*, which is successfully used for obtaining *spargel*^*del*^ cell clones (Fig. 2e–k). Upon induction of somatic clones in egg chambers and follicle cells, *srl*^*del/del*^ cell clones remain completely negative for Spargel expression (Fig. 2g) in nurse cells. This total lack of Spargel expression led us to conclude that we had created an amorphic *spargel* mutant, hence *srl*^*del*^ is considered as a *bona fide* null allele for the *srl* gene. Notably, we observed that 93% of *srl*^*null*^ egg chambers are trapped in the previtellogenic stages. Consistently, only a few *srl*^*null*^ egg chambers undergo vitellogenesis (yolk accumulation) and proceed to the postvitellogenic stage (Fig. 2d). Taken together, these data pinpoint that Spargel activity is more essential during later stages of oogenesis.

### Spargel RNA recognition motif (RRM) is functionally required in oogenesis

The three PGC-1 family members and their invertebrate homolog Spargel/dPGC-1 all carry an RNA recognition motif (RRM) and a RS domain in their C-terminal ends. *In vitro*, these motifs appear to confer RNA processing capacity (Monsalve *et al*. 2000), but it was not clear whether this was also the case in vivo. In subsequent studies, an engineered PGC-1α splice variant that is lacking both the RRM and the RS domains (NT-PGC-1α), was found to rescue all PGC-1α signature activities like mitochondrial biogenesis, adiposity and thermogenic capacity when expressed in PGC1α^−/−^ brown adipose tissue, where PGC-1α is highly expressed (Zhang *et al*. 2009; Chang *et al*. 2012). These findings are consistent with two mechanisms; either that the RRM and RS domains are not essential for PGC-1α function or that PGC-1α and/or PRC function mask the compromised activity of the engineered PGC-1α splice variant.

**Fig. 3.**
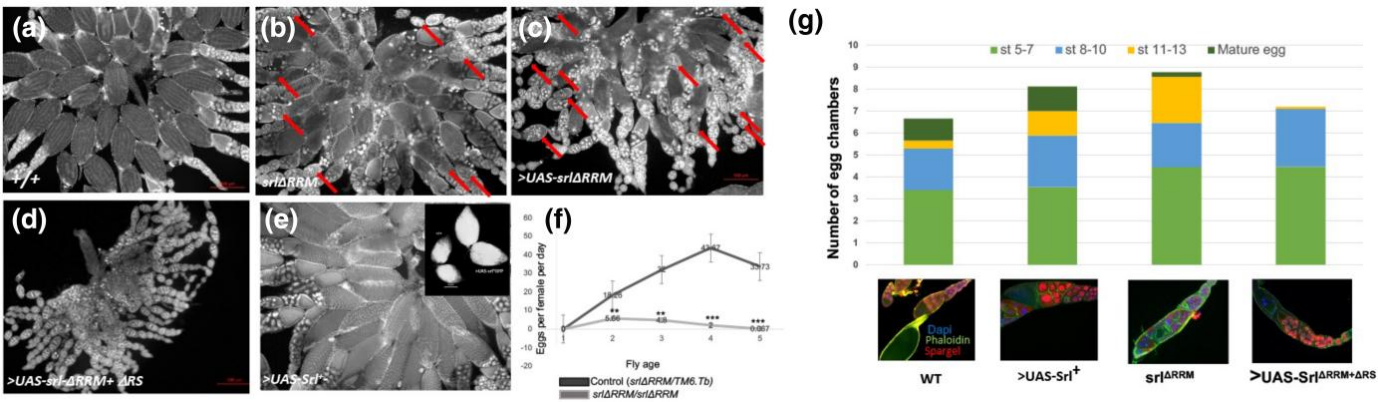
Influence of *spargel* mutant on oogenesis. Ovaries are prepared from 5 days old females that are kept in yeast supplemented media for 3 days before sacrifice. a) Wild type ovarioles with mature eggs, b) Endogenous *srl*^*>ΔRRM*^ ovaries are comparatively slower in post-vitellogenic development. Dumpless and apoptotic egg chambers (red arrows) are observed quite frequently, c) Overexpression of > UAS-*Srl^ΔRRM^* transgene slows down post-vitellogenic growth resulting in increased cell death, d) Transgenic > UAS-*srl*^*ΔRRM + ΔRS*^ overexpression cause no mature egg chambers in post-vitellogenic stages, e) Overexpression of wild type > UAS-*sr+* transgene enhances number of mature egg chambers in their ovaries representing a faster growth pattern. All transgene overexpression is driven by MTD-Gal4 driver. f) Comparison of egg laying between control and *srl*^*ΔRRM*^ females demonstrates that homozygous *ΔRRM* females produce virtually no eggs (g) Profiles of egg chamber numbers in growing ovaries. Compared to control Spargel overexpression produce a significantly large quantity of mature eggs as well as more egg chambers found in stage 8–13 (beginning of post-vitellogenesis) egg chambers. On the other hand, *srl*^*ΔRRM*^ ovaries are populated with stage 11–13 egg chambers although hardly few mature eggs are formed. Finally, transgenic overexpression of > UAS-*spargel*^*ΔRRM*^ and or > *UAS-Spargel^ΔRRM+ ΔRS^*proteins in wild type background shows severe mutant effect because no egg chambers can go past stage 8–10. n = 50 ovarioles are counted from 10 ovaries in each category. *P < 0.05, **P < 0.01, ***P < 0.001.

The primary structure of the RRM domains of vertebrate PGC-1 and *Drosophila* Spargel are quite comparable; both consist of 64% residues with 77 and 75 amino acids, respectively (Supplementary Fig. 1). Given that Spargel is the sole PGC-1 ortholog in insects (LeMoine *et al*. 2010; Mukherjee and Duttaroy 2013), we reasoned that reassessing the in vivo role of the RRM and RS domains in *Drosophila* could help us understand the extent to which these domains are required for PGC-1 function. Our data show that female homozygotes lacking the RRM domain (*srl*^*ΔRRM*^) produces virtually no eggs (Fig. 3f). Although oogenesis appears to proceed normally in *srl*^*ΔRRM*^ homozygous mothers until stage 8, postvitellogenic growth slowed down considerably, as evidenced by the accumulation of early egg chambers relative to controls (Fig. 3g). We also detected condensed and fragmented nurse cell nuclei and loss of filamentous actin, hallmarks of apoptosis (Soller *et al*. 1999; Phan *et al*. 2007), in a fraction of the *srl*^*ΔRRM*^ stage 8 or 9 egg chambers (Fig. 3b marked with arrow). Most of the *srl*^*ΔRRM*^ egg chambers that bypassed the stage 8 checkpoint failed to proceed to maturity (Fig. 3g). Taken together, these findings suggest that the RRM domain is required for progression of egg chamber development after vitellogenesis.

**Fig. 4.**
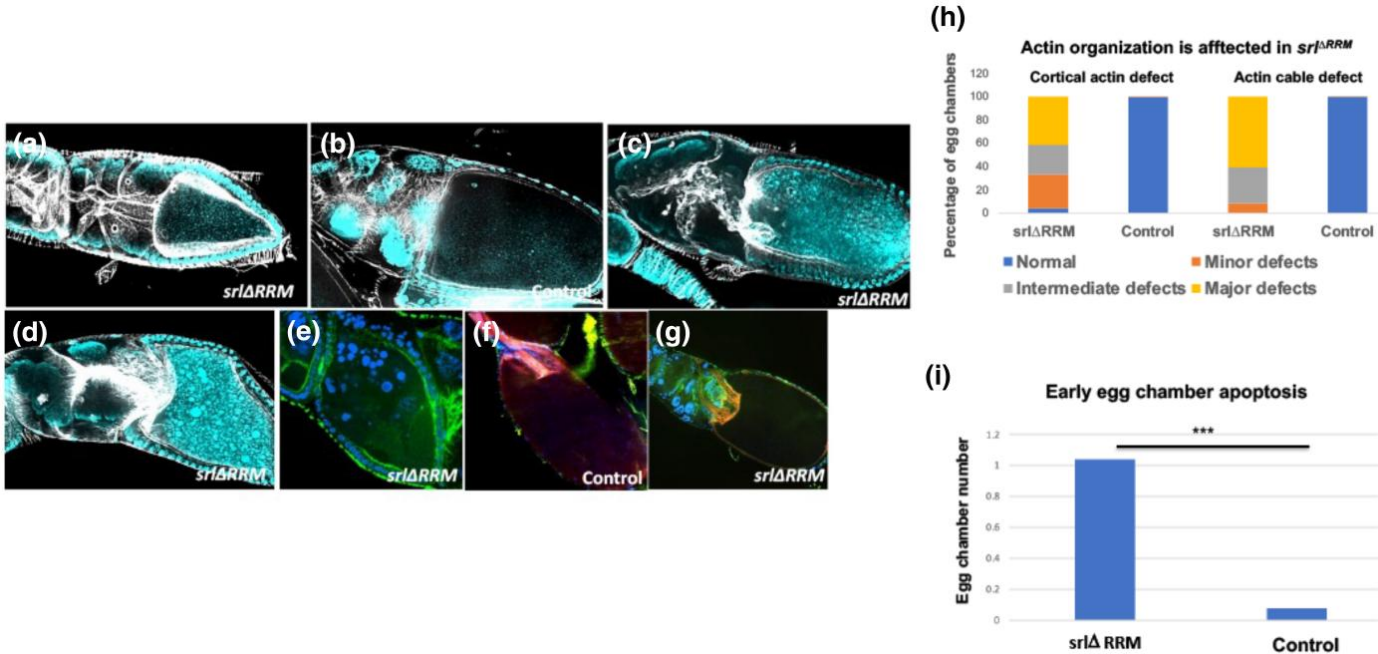
F-actin (phalloidin) staining displayed actin cable defects in *srl*^*ΔRRM*^ egg chambers. (a, c, d, e, g) *srl*^*ΔRRM*^ homozygote egg chambers. b, f) *Srl^ΔRRM^/+* (b) From stage 9 follicle cells undergo actin remodeling to form discernable actin cables extending from the nurse cell membrane towards the nuclei, a) actin cable distribution appears normal in *srl*^*ΔRRM*^ egg chambers until stage 8, however variety of defects appeared from stage 9 onwards such as fused actin cables (c) two or more nurse cell nuclei in stage 9 egg chamber with disrupted actin cable formation as well as cortical actin breakdown (d), post-vitellogenic egg chambers undergoing apoptosis with nurse cell nuclear fragmentation and dissociation of nurse cell and oocyte cortical actin (e). f) In stage 11 egg chambers contract to transfer their cytoplasmic content into the oocyte, an event known as dumping. g) dumping defects in *srl*^*ΔRRM*^ egg chambers associated with retention of substantial amount of nurse cell cytoplasm, h) Quantification of actin defect seen in *srl*^*ΔRRM*^ stage 10 egg chambers. Stage 10a: n = 24; Stage 10b: n = 23, i) Quantification of early egg chamber death in *srl*^*ΔRRM*^. n = 25. *** *P* ≤ 0.001.

At the onset of stage 10b, nurse cells execute a process known as “dumping”, this active process is critical for nuclear positioning in which a cage of actin cables that hold the nurse cell nuclei in position with respect to the cortical actin begins to form (Cooley *et al.* 1992). To investigate whether actin dynamics in nurse cells was impacted in *srl ^ΔRRM^* flies, we stained the actin cytoskeleton with a Phalloidin derivative. Although nurse cell actin looked no different from control stage egg chambers until stage 8 (Supplementary Fig. 2), from stage 9 onwards, cortical actin in *srl*^*ΔRRM*^ egg chambers was weakened or broken down, and two or more nurse cell nuclei were often bunched together with no nurse cell membrane separating them (Fig. 4c). This phenotype was highly distinct from the thickening of cortical actin in control egg chambers characteristic for this stage (Tootle and Spradling 2008).

In stage 10 egg chambers in control embryos, we detected the expected robust cage of actin cables as well as strong cortical actin staining (Fig. 4b) but found weak, partial, or missing cortical actin and actin cable formation in more than 90% of *srl^ΔRRM^* embryos (Fig. 4h and Supplementary Figs. 3 and 4). Consequently, there is no dorsal appendage formation but an incomplete chorion, such as the ones with cup-shaped eggs, in *srl^ΔRRM^* egg chambers (Fig. 4g). As these eggs have little or no visible dorsal appendage, they cannot be staged. Based on these data, we conclude that the lack of the cage structure necessary for actin-mediated contractile force severely inhibits the dumping process in *srl ^ΔRRM^* egg chambers starting from stage 10b till stage 14.

### Induced overexpression of wild type Spargel and *Spargel^ΔRRM^* has opposing effects on oogenesis

GAL4 induced overexpression of a wild type *spargel* (>*UAS-srl*^*+*^*GFP*) in the ovary, caused accumulation of an impressively large number of post-vitellogenic egg chambers (Fig. 3e) relative to wild type (Fig. 3a). Although the presence of excess Spargel is associated with larger ovaries compared to control (Fig. 3g inset), high amounts of Spargel does not appear to have any toxic effects, but purely expedites the growth of egg chambers. In contrast, induced overexpression of the Sparge*l^ΔRRM^* protein (>*UAS- srl ^ΔRRM^, GFP)* (Fig. 1) had significant negative effects on oogenesis. Given excess cell death, no postvitellogenic egg chambers had formed (Fig. 3c and d). In a more extreme situation, combined induction of SrlΔRRM + ΔRS protein cause totally rudimentary ovaries to form (Fig. 4d). Based on these data, we conclude that overproduction of truncated Spargel proteins overpower the activity of wild type Spargel in a dominant-negative manner. However, additional experiments would be needed to fully understand the dominant negative effect of SpargelΔRRM.

### Aggregation of Notch and Delta in *spargel* mutants is related to impaired cellular trafficking

**Fig. 5.**
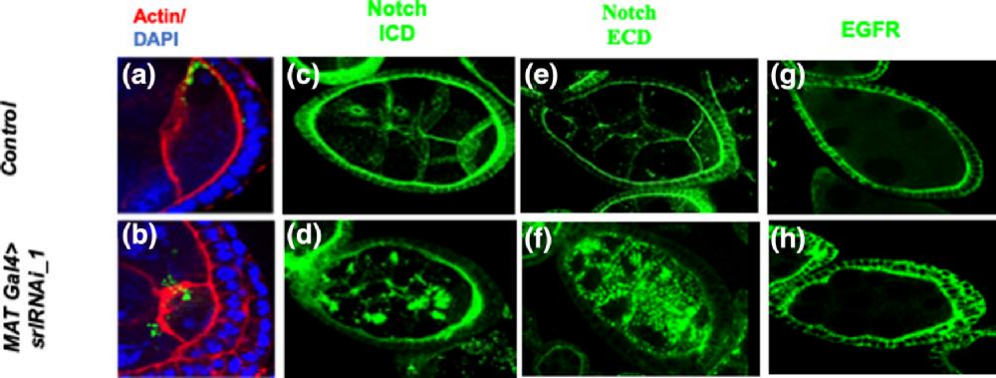
Notch aggregates in *spargel* knockdown germline. a, b) Multiple layering of the follicle cells in *MATGal4 > srlRNAi_1* egg chamber. F-Actin (phalloidin), Red; DNA (DAPI) blue; c, d) Notch is mostly expressed at the boundary of follicle cells in control egg chambers, however *MATGal4 > srlRNAi_1* egg chamber displays large aggregates of Notch, in the form of very large punctate, inside nurse cell cytoplasm. Notch expression is detected with a primary antibody against the intracellular domain (ICD) of Notch (green). e, f) Primary antibody against the extracellular domain (ECD) of Notch detected similar aggregates of Notch in *MATGal4 > srlRNAi_1* egg chamber with a slightly higher frequency than the ICD. g, h) EGFR, another follicle cell membrane protein, does not aggregate in *MATGal4 > srlRNAi_1* egg chamber. t-test was performed to determine the significance. Scale bar = 50 μm.

Stage 6 germ cells activate Notch in follicle cells as a mechanism for inhibiting mitosis of the follicular epithelium (Deng *et al*. 2001; López-Schier and St. Johnston 2001), and that failure to activate Notch signaling leads to follicle cell over-proliferation (López-Schier and St. Johnston 2001). We observed multilayering of follicle cells in *MAT Gal4 > srlRNAi_1* egg chamber upon *srl* knock down (Fig. 5a and b), so we considered that disrupted Notch signaling might explain this phenotype. Notch is expressed in follicle cells at the inner edge of the follicle/germ cell boundary in wild-type flies (Fig. 5c). However, in *MAT Gal4 > srlRNAi_1* egg chamber, we detected strikingly large Notch protein aggregates, shaped like conspicuously large puncta, that were distributed randomly throughout the nurse cell cytoplasm (Fig. 5d). Unlike Notch, EGFR did not form aggregates in the *MAT Gal4 > srlRNAi_1* egg chamber (Fig. 5, g and h). Thus, the aggregation of Notch in the germ cell cytoplasm appears to be unique, and not a general feature of all transmembrane proteins.

**Fig. 6.**
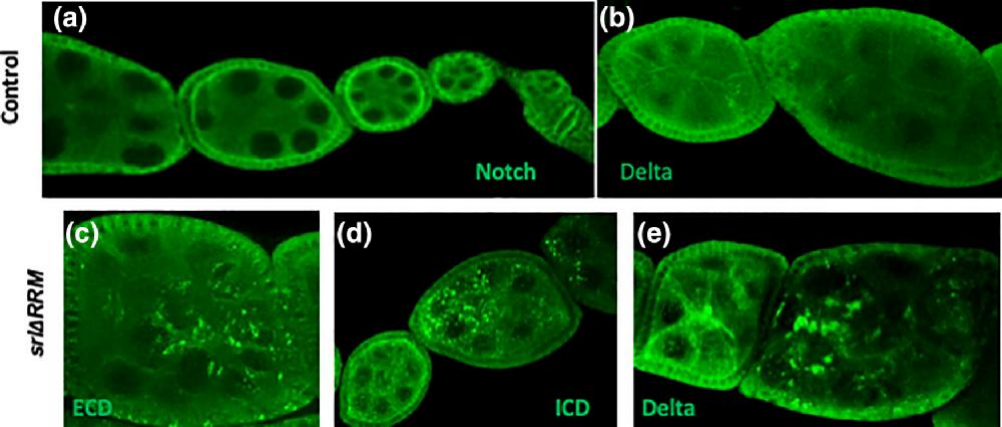
Notch aggregates appear in endogenous *spargel* mutants. a) Notch and Delta expression in control egg chambers. c–e) Aggregates of Notch and Delta appears in *srl*^*ΔRRM*^ egg chambers.

Notch Extra Cellular Domain (ECD), Intracellular Domain (ICD) and Delta all appear to form puncta in the nurse cell cytoplasm of stage 6–8 *srl ^ΔRRM^* egg chambers, albeit rather close to the nurse cell membranes (Fig. 6a–e). In general, it appears that Notch ECD and ICD intensities at the follicle/nurse cell boundary seem to be decreased in the *srl ^ΔRRM^* stage 6–8 egg chambers compared to controls, which is expected given the increase in cytoplasmic Notch ECD punctae. The binding of the Notch ECD to the transmembrane Delta ligand and subsequent trans-endocytosis is critical for Notch receptor activation and subsequent Notch signaling (Shaya *et al*. 2017; Heuss, *et al*. 2008; Nichols *et al*. 2007; Parks *et al*. 2000). Following trans-endocytosis, the Notch ECD is either recycled back to the membrane or transported to other endocytic compartments, including multivesicular bodies/late endosomes and lysosomes (Fortini and Bilder 2009). Therefore, disruption in endocytic cellular transport can lead to accumulation of the Notch ECD in the signal-sending cell, as observed in nurse cells (Vaccari and Bilder 2005).

**Fig. 7.**
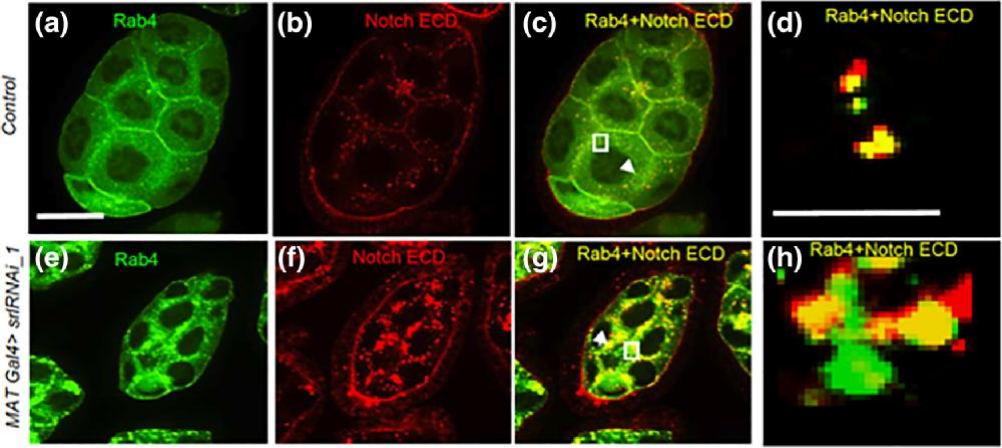
Loss of Spargel affects cellular trafficking (a, b) In control egg chambers, Rab4 and Notch are diffusely distributed throughout the cytoplasm. c) Merge shows the appearance of tiny yellow punctae throughout the cytoplasm (arrowhead), confirming Notch and Rab4 co-localization (d) A magnified view (taken from image C which is shown as square) confirmed that Notch and Rab4 are often colocalized in the form of tiny punctate structures. e, f) Both Rab4 and Notch accumulated in the form of large aggregates in *MATGal4 > srlRNAi_1* egg chamber. g) Merge of Notch and Rab4 staining showed that they are present in the large aggregates (marked as 1 with arrow) found in *spargel* depleted egg chambers (h) A magnified view (taken from image G which is shown as square) of the Notch and Rab4 aggregates appears much larger than the control. Control genotype is *srlRNAi* line without Gal4 drivers. Scale bar, a–g 50 μm and d, h 2.5 μm.

Ras-associated binding proteins (Rab) play central roles in intracellular Notch transport (Stenmark 2009). Specifically, Rab4 regulates endocytosis of Notch and other membrane proteins (Van Der Sluijs *et al*. 1992; Gomez-Lamarca *et al*. 2015). To investigate whether Spargel depletion affects endosomal trafficking, we monitored the subcellular localization of Rab4 and Notch by immunohistochemistry. We observed co-localization of large Rab4 granules and Notch aggregates in *MAT Gal4 > srlRNAi_1* ovarioles (Fig. 7e–g). Live imaging of Rab4-GFP in egg chambers revealed many mobile, tiny punctate structures in control and *MAT Gal4 > srlRNAi_1* embryo (Supplementary Videos 2 & 3). However, larger Rab4-GFP aggregates were also present in *MAT Gal4 > srlRNAi_1* egg chamber (Fig. 7h). Upon careful observation of time lapse image we found that the punctae are changing their position over time in control embryos but not in *MAT Gal4 > srlRNAi_1*, where they remain static. Thus, loss of Spargel impairs endocytic transport.

### Depletion of the nutrient sensor TOR triggers Notch aggregation

**Fig. 8.**
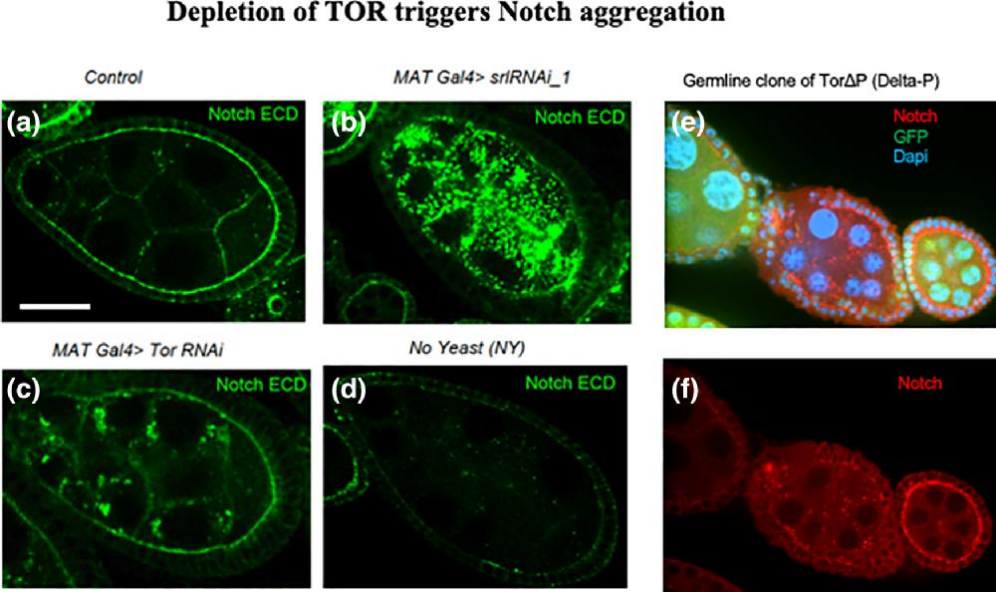
Depletion of TOR triggers Notch aggregation. a) Notch ECD staining showed Notch is expressed at the apical surface of the follicle and nurse cell cytoplasmic membrane. b) Aggregates of Notch appears in Spargel-depleted germ cells. c) Notch aggregation was also noted in clones of TOR (Target of Rapamycin)-depleted egg chambers. d) No aggregated Notch appeared in egg chambers following starvation. e, f) Aggregation of Notch in germline clones of TorΔP (e) merged (f) Notch. Control genotype is *srlRNAi_1* line without Gal4 drivers. Scale bar, 50 μm.

The Target of Rapamycin (TOR) is a well-known nutrient sensor. Earlier studies in *Drosophila* suggested that TOR acts upstream of Spargel in the insulin signaling pathway (Mukherjee and Duttaroy 2013) and that inactivation of TOR in the ovary, like loss of Spargel, causes oogenesis defects leading to infertility (Zhang *et al*. 2006). In addition, TOR has been reported to be involved in endocytic transport (Hennig *et al*. 2006; Tenay *et al*. 2013). Here, we asked whether knockdown of TOR would recapitulate the Notch aggregation phenotype in Spargel-depleted embryos. Indeed, *MAT Gal4 >TOR RNAi* egg chambers displayed cytoplasmic aggregation of Notch, though to a lesser extent than *MAT Gal4 > srlRNAi_1* egg chamber (Fig. 8a–d). Thus, the fact that Notch aggregation is evident in TOR depleted cell clones confirms that endocytic transport is negatively affected through TOR as well as Spargel depletion (Fig. 8e). In contrast, yeast withdrawal did not induce Notch aggregation in the egg chambers (Fig. 8d). Taken together, these data support that TOR and Spargel influences Notch aggregation and endocytic trafficking independent of nutrient signaling.

## Discussion

### New mutant alleles of *spargel* are yielding additional biological role for this protein

As revealed in this report, newly generated *srl* mutant alleles are shedding more light on the functional diversity of the ancestral form of PGC-1 in invertebrates. The necessity of the RNA Recognition Motif (RRM) in a PGC-1 group of proteins is established for the first time. Hence *srl*^Δ^*^RRM^* mutant flies could be used to investigate protein/RNA and RNA/RNA interactions and discover binding specificity of the RRM domain. Given that the RRM domain is conserved between PGC-1 partners, it's conceivable that assessing its functionality and binding partners in *Drosophila*, free of interference from other PGC-1 orthologs, could help us understand RRM function in vertebrates including humans. Thus, the insights gained from studying the *srl* mutants described here broadens the role of *Drosophila* Spargel beyond female germline development to essential roles in embryogenesis and somatic tissues. Investigations into the requirement of Spargel function in developing somatic tissues such as salivary gland, muscle, and intestine are currently underway (Duttaroy lab, in preparation).

Earlier studies using RNAi knockdown and the hypomorphic *sparge*l allele (a.k.a *srl*^*1*^) established the importance of Spargel in oogenesis (Basar *et al.* 2019), growth, fertility (Basar *et al.* 2019), adult survival (Mukherjee *et al* 2014) and with the Insulin Signaling pathway (Mukherjee *et al*. 2014). However, the limited to no capacity of a hypomorphic and or knock down allele in establishing the cell autonomous function of a protein is already known for which a null mutant allele is appropriate. Through the establishment of a *srl*^*null*^ allele opens the opportunity to test in vivo interaction of Spargel with other cellular proteins and how that regulates multitude of biological processes.

### Within the PGC-1 gene family, Spargel and PRC shares most functional similarities

**Fig. 9.**
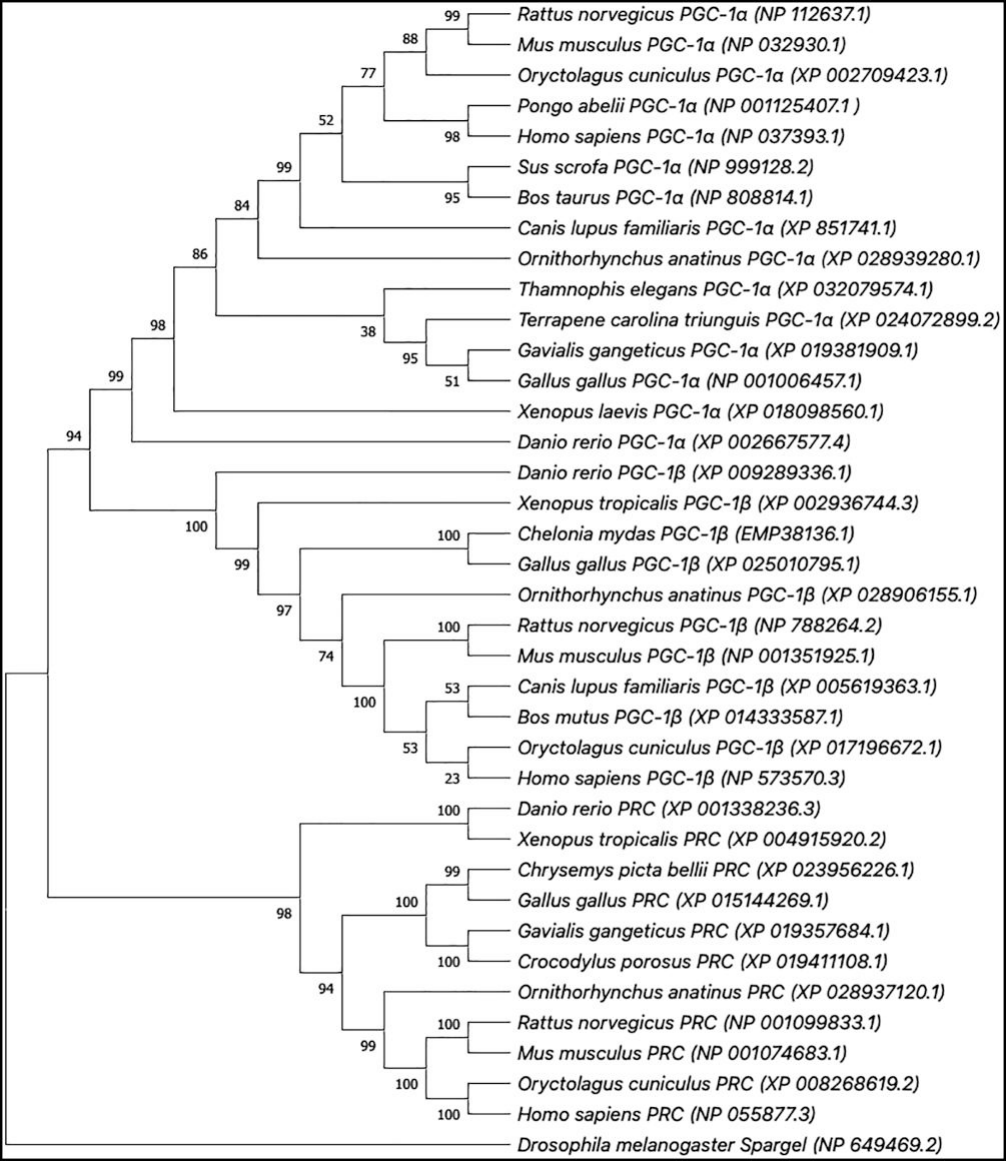
Phylogenetic tree of the PGC-1 family including Spargel. Phylogenetic tree of the PGC-1 family including *Drosophila* Spargel. The tree was constructed based on protein sequences using maximum likelihood approach of MEGA X. All the sequences were retrieved from NCBI with accession numbers mentioned in parentheses. All nodes were supported by more than 90% except some.

Prior homology-based analysis revealed that *Drosophila* PGC-1-like protein (CG9809) is orthologous to mammalian PGC-1 s because they share significant structural similarities, including various domains (Gershman *et al*. 2007). Phylogenetic investigation of vertebrate PGC-1 s with insect PGC-1-like proteins unmasked that the PGC-1-like protein in *Drosophila* represents the ancestral form (LeMoine *et al*. 2010), where they used software-translated protein sequences (using *Drosophila* codon bias) derived from nucleotide sequences. Later, the PGC-1 like protein in *Drosophila* was identified as the endogenous Spargel protein (Tiefenböck *et al*. 2010). We retrieved the most updated amino acid sequences of *Drosophila* Spargel and other vertebrate PGC- 1 s from the National Centre for Biotechnology Information (NCBI) and used these protein sequences as our input to construct a phylogenetic tree to determine the evolutionary relationship between these proteins (Fig. 9). Our evolutionary analysis has confirmed Spargel as the ancestral form of vertebrate PGC-1 because the tree demonstrate that all the branches originate from the Spargel node (Fig. 9). Taken together, we conclude that both nucleotide and amino acid-based phylogenetic analysis support the *Drosophila* Spargel protein is the ancestral form of vertebrate PGC-1 s.

Among PGC-1 family members, Spargel and PRC shares many functional similarities, based on the following facts: (1) PRC gene silencing negatively influences growth of somatic cells (Andersson and Scarpulla 2001) similar to *spargel* knockdown where growth retardation is the primary event (Basar *et al.* 2019), (2) A BLAST search with human PGC-1's and Spargel reveals higher similarity scores between PRC and Spargel proteins (Supplementary Table 1), (3) germ-line knockouts of *PRC* resulted in peri-implantation embryonic lethality (He *et al*. 2012), similar to the late embryonic/early larval lethality observed in *Drosophila spargel* loss of function, and (4) both *srl* and *PRC* are induced under high nutrient condition; while *PRC* is induced in quiescent cells following serum addition (Vercauteren *et al*. 2006) like the *Drosophila spargel* which is highly induced in nutrient enriched media (Gershman *et al*. 2007; Basar *et al.* 2019).

### How might Spargel affect the Actin cytoskeleton?

Phenotypes of *srl*^Δ^*^RRM^* mutant indicates some kind of major effect during transition to vitellogenesis at around stage 8/9 when the egg chambers go through a developmental checkpoint (Terashima and Bownes 2004). Lack of RRM domain appears to be altering either steroid signaling or prevent lipid deposition in the ovaries or both since stage 8/9 developmental checkpoint-induced apoptosis can be activated by both (Buszczak *et al*. 2002; Parra-Peralbo and Culi 2011). As mentioned earlier, the severe defects in actin cytoskeleton in *srl*^Δ^*^RRM^* mutant likely impairs the dumping process which is essential for female fertility (Cooley *et al.* 1992; Huelsmann *et al*. 2013).

All mutants of actin regulators have *dumpless* defects and they all are female sterile (Cooley *et al.* 1992; Hudson and Cooley 2002; Zallen *et al*. 2002; Verdier *et al*. 2006; Spracklen *et al*. 2019).

Another possibility is prostaglandins which participate in the regulation of the dumping process. Previous studies confirmed that the *Drosophila* Cyclooxygenase enzyme Pxt is required for both the parallel actin filament bundle formation and the cortical actin strengthening essential for dumping (Groen *et al*. 2012). Indeed, *Pxt* hypomorphic mutants show dumping defects associated with defective actin cytoskeleton, much like the *srl ^ΔRRM^* mutants (Tootle and Spradling 2008). Not only do *Pxt* mutants display actin cable defects, but they also show loss of cortical actin integrity and breakdown of nurse cell membrane (Tootle and Spradling 2008). Therefore, it is highly probable that Spargel regulates the actin cytoskeleton via lipid or prostaglandin signaling.

### Current findings support intracellular transport of Notch is inhibited in *srl* mutants

Notch signaling plays a prominent role in *Drosophila* oogenesis as well as in the mammalian ovary (Xu *et al*. 1992; Larkin *et al*. 1996; Grammont and Irvine 2001; Vanorny and Mayo 2017). In *Drosophila* germline function for Notch is not known, however germline Delta signaling is required to activate follicular Notch which in turn arrest mitosis in stage 6 egg chambers. Thus, appropriate contact between germ cells and follicle cells is essential to maintain proper intercellular communication. Spargel depletion does not seem to impact on this contact but nevertheless leads to loss of Notch signaling in follicle cells, likely due to Notch aggregation in germ cell cytoplasm. Earlier studies support that the Notch ECD, which is trans-endocytosed into the signal-sending cell and recycled back to the membrane, plays a critical role in the Notch signal receiving cell (Shaya *et al*. 2017; Heuss, *et al*. 2008; Nichols *et al*. 2007; Parks *et al*. 2000). Therefore, disruption of Notch ECD endocytosis or recycling can potentially affect Delta-mediated Notch signaling in follicle cells. Furthermore, alterations in the endocytic pathway can trigger co- aggregation of Notch and Rabs (Gomez-Lamarca *et al*. 2015), like what we observed following Spargel knockdown. Live cell imaging combined with immunohistochemistry suggest that the enlarged granules corresponding to Notch and Rab4 are immobile. We infer that the core pathway for endocytosis or receptor recycling requires the action of Spargel, either directly or indirectly. Since endocytic trafficking is an energy-dependent process, the decreased mitochondrial numbers in *MAT Gal4 > srlRNAi_1* germ cell might explain the impaired endocytic trafficking observed in these egg chamber. Based on previously reported gene array analysis, Spargel regulates more than 50% of small-GTPase-mediated signal transduction and vesicle-mediated transport related genes (Tiefenböck *et al*. 2010). A recent report also found that PGC-1 inhibits endothelial migration by activating Notch signaling (Sawada *et al*. 2014). Additionally, the involvement of TOR in endocytic transport and as a mediator of cell growth is evident (Hennig *et al*. 2006; Tenay *et al*. 2013). Moreover, it has been reported that dietary lipid regulates somatic cell proliferation in a Notch signaling-dependent manner (Obniski *et al*. 2018). Hr96 (a ligand that binds cholesterol) mutants show same phenotype of Notch and delta aggregation in the germ cell cytoplasm (Obniski *et al*. 2018). Taken together, our findings provide insight into cellular processes regulated by Spargel and provide a framework for identifying and investigating its intracellular partners/targets to clarify its mechanistic role in endocytic trafficking and Notch signaling.

## Supplementary Material

jkad142_Supplementary_Data

## Data Availability

Strains and plasmids are available upon request. The authors affirm that all data necessary for confirming the conclusions of the article are present within the article, figures, and tables. Supplemental material available at G3 online
