# Supplementary material for: New mutant alleles for Spargel/dPGC-1 highlights the function of Spargel RRM domain in oogenesis and expands the role of Spargel in embryogenesis and intracellular transport
Source: G3 (Bethesda). 2023 Jun 27;13(9):jkad142. doi: 10.1093/g3journal/jkad142 (PMC10468312; doi:10.1093/g3journal/jkad142)
Supplement: jkad142_Supplementary_Data [file jkad142_supplementary_data.zip › Supplemental_Figures_S1-S4_and_Supplemental_Table_S1_G3-2023-404307.pdf]

Supplemental Figure S1

Sequence ID: **Query_22005** Length: **77** Number of Matches: **1**

Range 1: 1 to 77 Graphics ▼ Next Match ▲ Previous Match

Score	Expect	Method	Identities	Positives	Gaps
60.1 bits(144)	6e-19	Compositional matrix adjust.	28/77(36%)	50/77(64%)	2/77(2%)

```
Query  1   RIVYVGRIEQETTKEILRRKFLPYGSIKQITIHYKENGMKYGFVTYERAQDAFTAIDTSH  60
           R++YVG+I  +TT+  LR +F  +G I++ T++ +++G  YGF+TY    DAF A++  +
Sbjct  1   RVIYVGKIRPDTTRTELRDRFEVFGEIEECTVNLRDDGDSYGFITYRYTCDAFAALENGY  60

Query  61  --RDSQISMYDISFGGR  75
             R S  + +++ F GR
Sbjct  61  TLRRSNETDFELYFCGR  77
```

Query= *spargel* RRM (Drosophila)
Sbjct= *PGC-1* RRM (Mammal)

Supplemental Figure S2

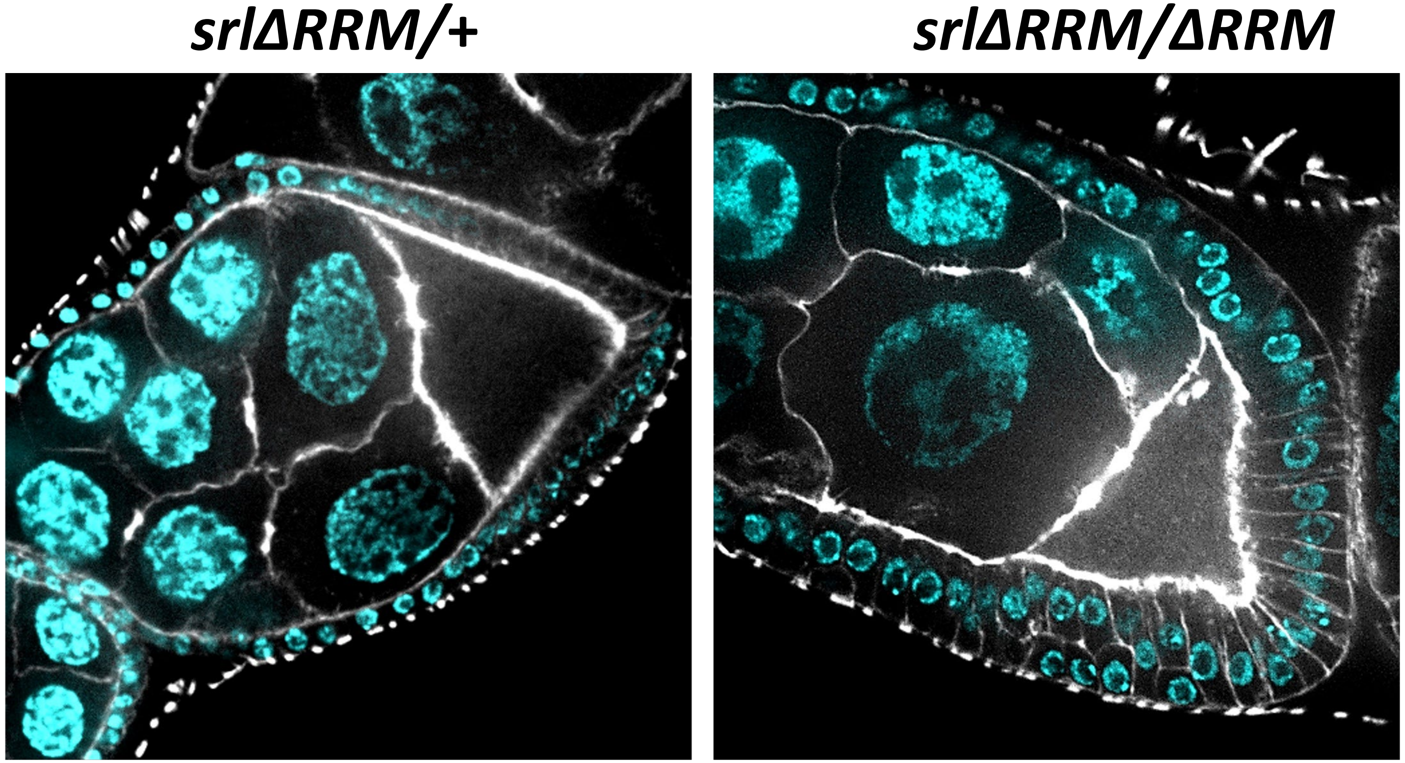

Stage 8

Supplemental Figure S3

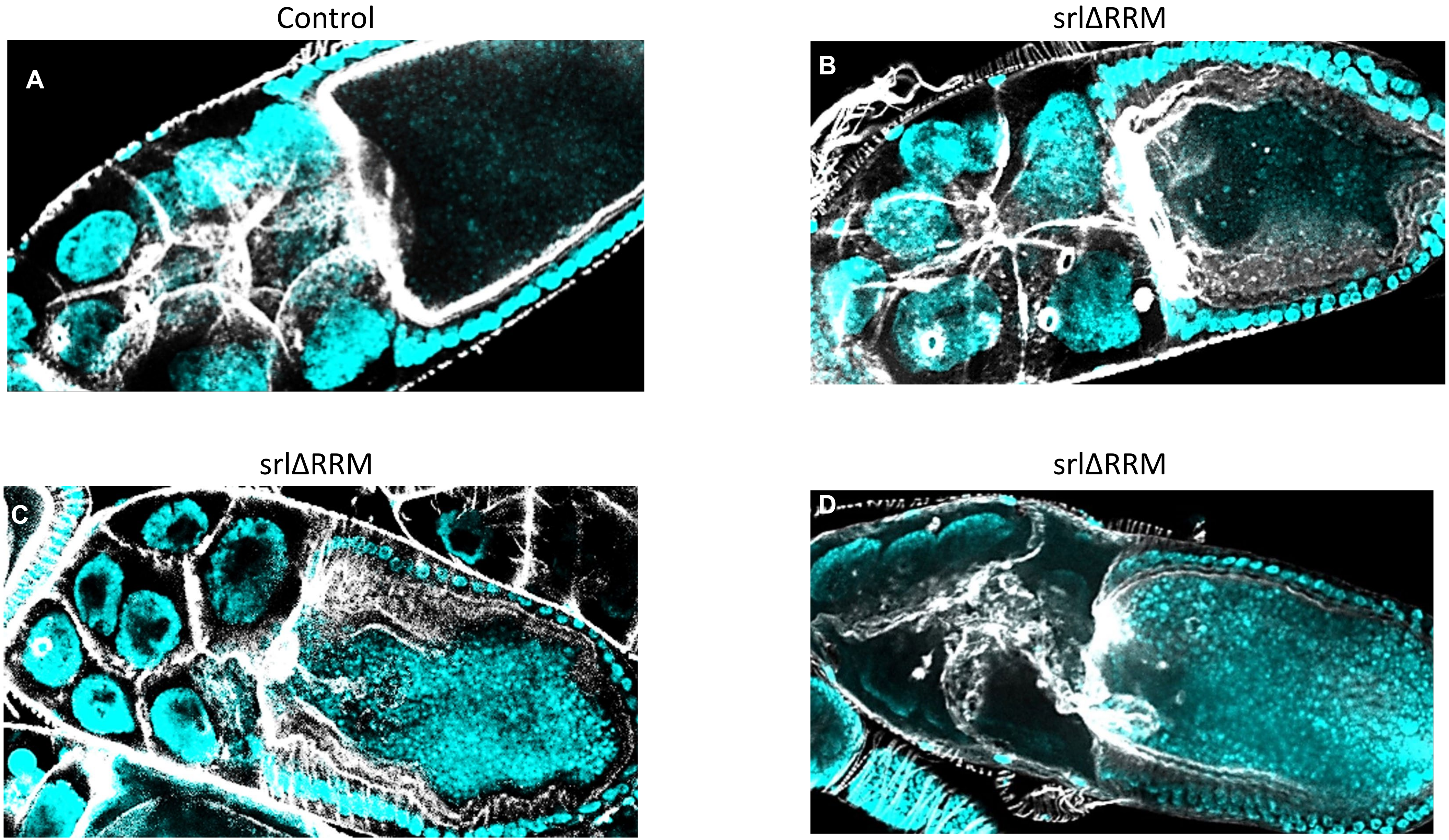

Supplemental Figure S4

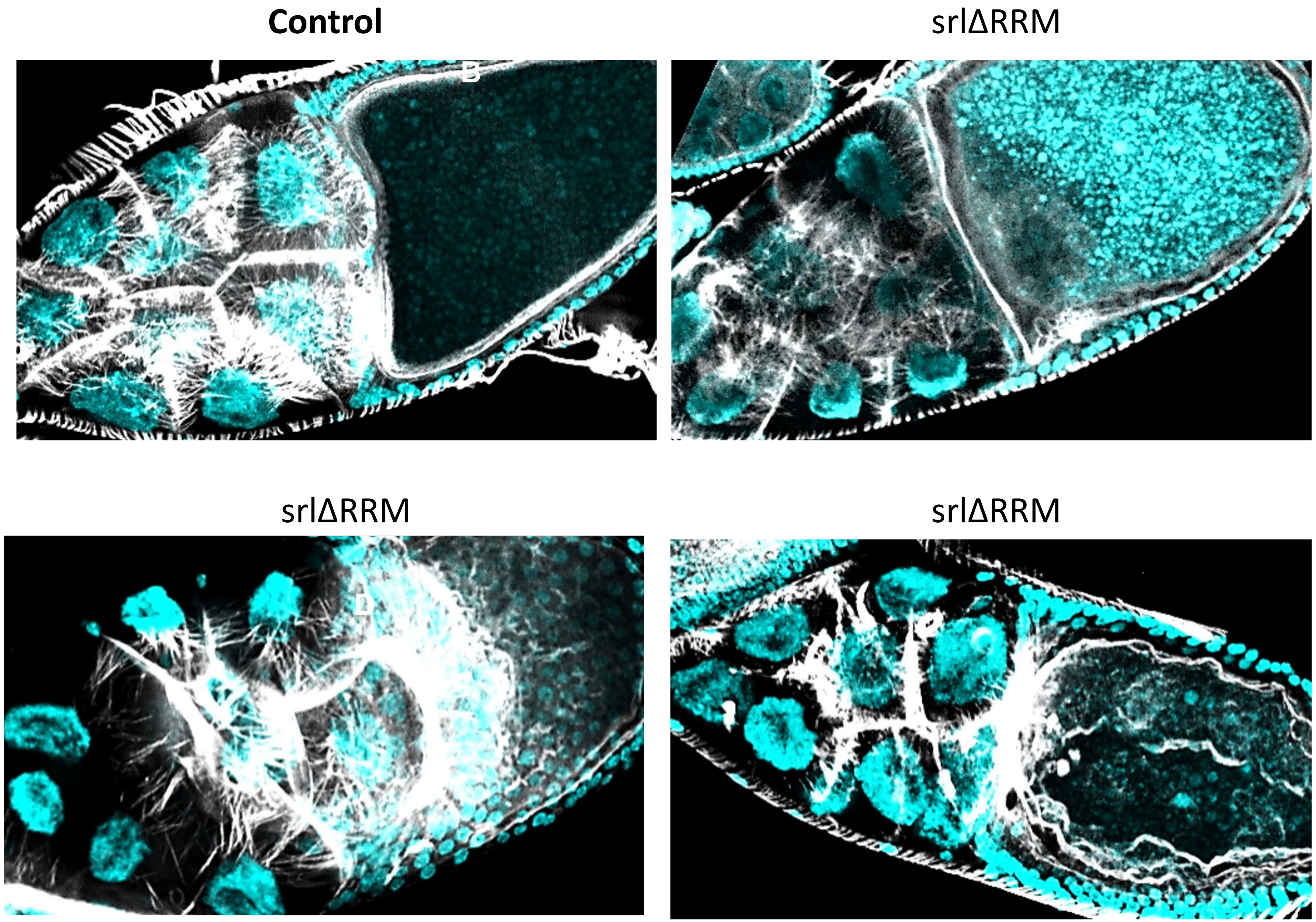

Supplemental Table S1

**Characteristics**	**HumanPGC-1alpha vs.Spargel**	**HumanPGC-1 beta vs.Spargel**	**HumanPRC-1 vs.Spargel**
E-value	$1e^{-22}$	$1e^{-12}$	$2e^{-27}$
Percent identity	41.25%	29%	45%
Query cover	41%	11%	
