# Supplementary material for: New mutant alleles for Spargel/dPGC-1 highlights the function of Spargel RRM domain in oogenesis and expands the role of Spargel in embryogenesis and intracellular transport
Source: G3 (Bethesda). 2023 Jun 27;13(9):jkad142. doi: 10.1093/g3journal/jkad142 (PMC10468312; doi:10.1093/g3journal/jkad142)
Supplement: jkad142_Supplementary_Data [file jkad142_supplementary_data.zip › Supplemental_Material_Legends_G3-2023-404307.pdf]

SUPPLEMENTAL MATERIAL LEGENDS

Supplemental Figure S1: RRM domains of PGC-1 and Spargel. BlastP results between query Spargel RRM (*Drosophila*) and subject PGC-1 RRM (Mammal) shows there is 36% identity with 64% positive hits and a low number (2%) of gaps indicating similarity between the two domains.

Supplemental Figure S2: $srl^{\Delta RRM/\Delta RRM}$ cortical actin strength, integrity, and distributions are very similar to controls prior to stage 8.

Supplemental Figure S3: Examples of Cortical actin defects in $srl^{\Delta RRM/\Delta RRM}$ stage 10a. Observed actin defects are classified into three categories: 1. Minor defects, 2. Intermediate defects, 3. Major defects. **(A)** In wild type actin is localized in the plasma membrane. **(B)** Minor defects: one nurse cell contains two nuclei surrounded by cortical actin. **(C)** Intermediate defects: Two or more nuclei are present in one

nurse cell. **(D)** Major Defects: Multinucleated nurse cells are observed with significantly reduced cortical actin.

Supplemental Figure S4: Actin cable defects in *srl*$^{\Delta RRM/\Delta RRM}$ stage 10b. (A) In control stage 10b Actin bundles connect the nucleus and plasma membrane forming actin cables throughout the cytoplasm. **(B)** Minor defect: Decreased density of actin cables are observed in otherwise normal egg chambers. **(C)** Intermediate defects: Significantly decreased and or lost actin cables. **(D)** Major defects: Extremely short or absent actin cables.

Supplemental Table S1

BlastP search among PGC-1 members and Spargel reveals that PRC is closest.
