# Supplementary material for: New mutant alleles for Spargel/dPGC-1 highlights the function of Spargel RRM domain in oogenesis and expands the role of Spargel in embryogenesis and intracellular transport
Source: G3 (Bethesda). 2023 Jun 27;13(9):jkad142. doi: 10.1093/g3journal/jkad142 (PMC10468312; doi:10.1093/g3journal/jkad142)
Supplement: jkad142_Supplementary_Data [file jkad142_supplementary_data.zip › Supplemental_Videos_S1-S3_G3-2023-404307.pptx]

## Slide 1
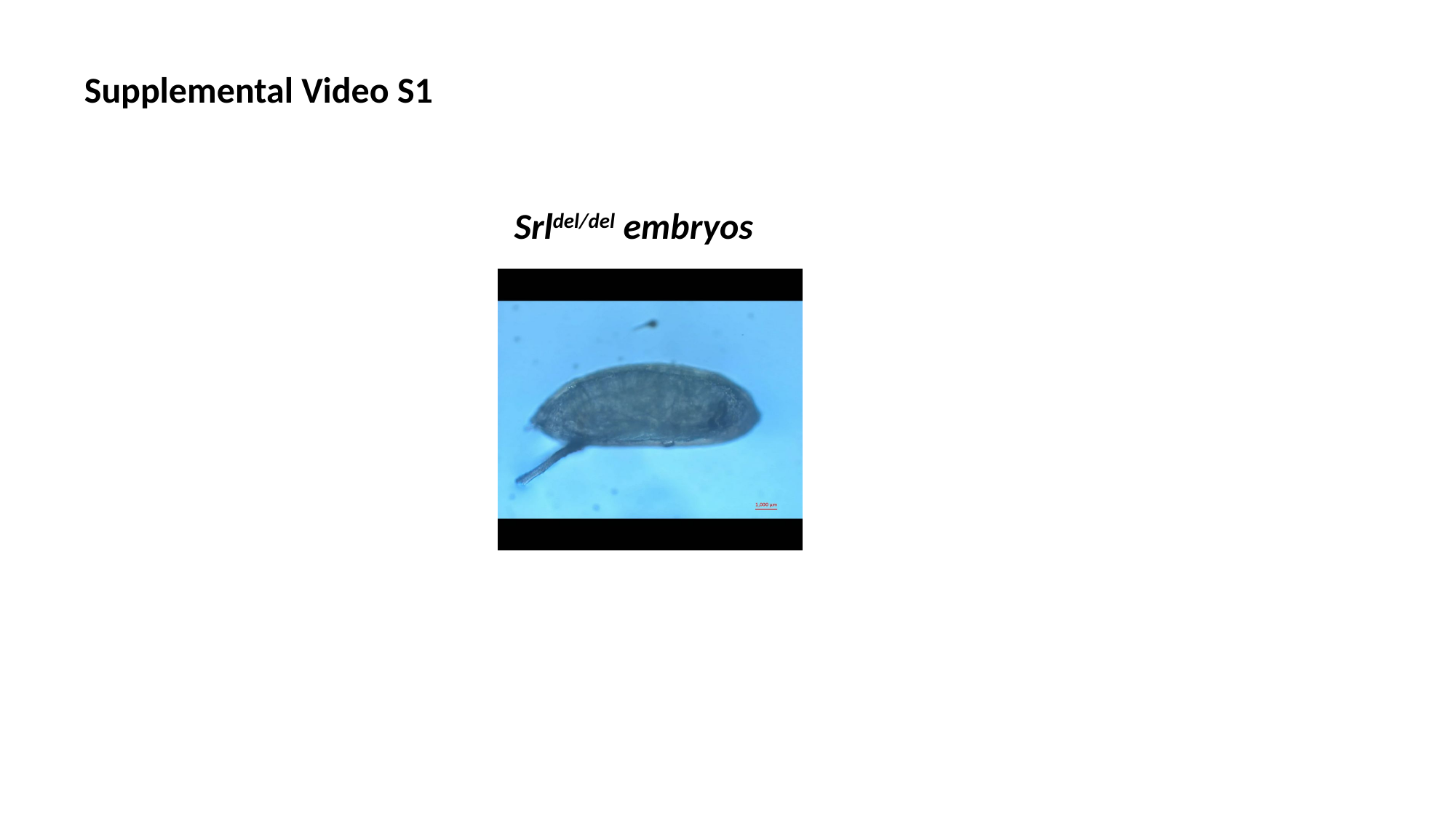

Supplemental Video S1
Srldel/del embryos

## Slide 2
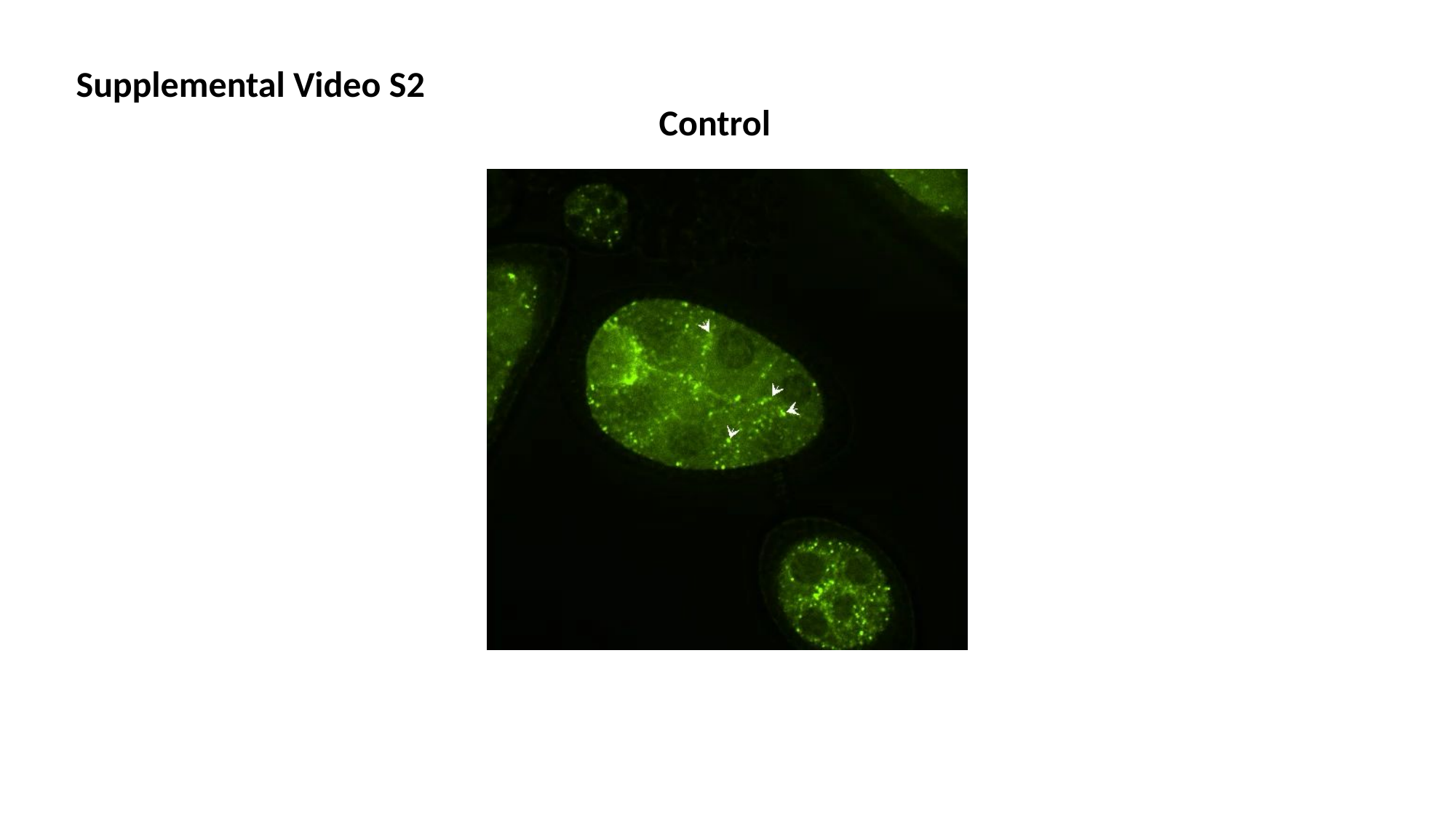

Supplemental Video S2
Control

## Slide 3
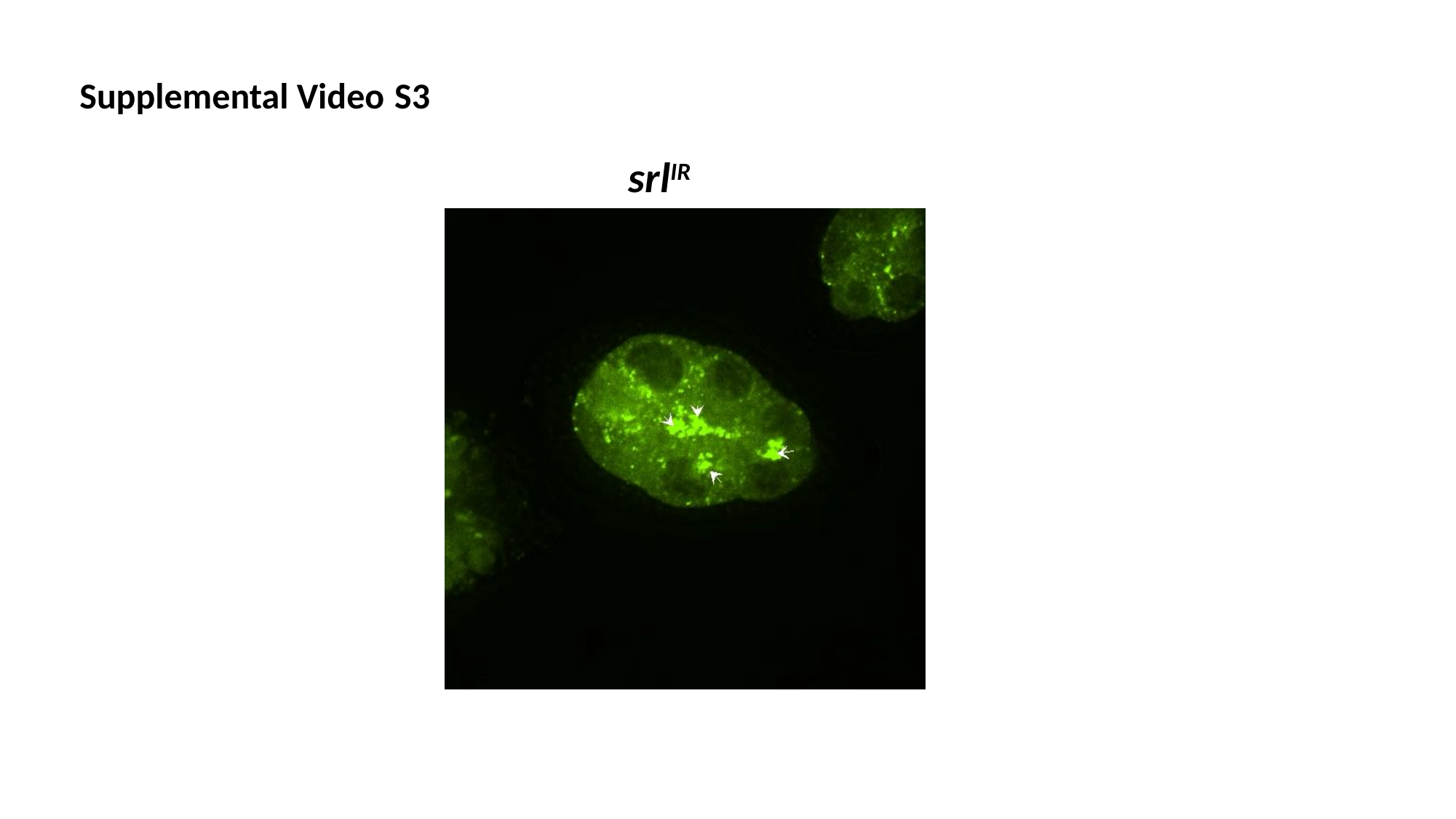

Supplemental Video S3
srlIR
